# Recurrent Acute Liver Failure in a Bahraini Child With a Novel Mutation of Spinocerebellar Ataxia-21

**DOI:** 10.7759/cureus.36249

**Published:** 2023-03-16

**Authors:** Hasan M Isa, Jawaher F Alkaabi, Wasan H Alhammadi, Khadija A Marjan

**Affiliations:** 1 Department of Pediatrics, Arabian Gulf University, Manama, BHR; 2 Department of Pediatrics, Salmaniya Medical Complex, Manama, BHR

**Keywords:** bahrain, spinocerebellar ataxia 21, scyl1, genetics, acute liver failure, child

## Abstract

Acute liver failure (ALF) in children is a rare life-threatening condition. ALF is caused by different etiologies. The most common causes are drug-induced liver injury, infections, and metabolic diseases. Other rare causes of ALF are genetic disorders including spinocerebellar ataxia-21 (SCAR21). Herein, we describe the first Bahraini child who was diagnosed with a novel homozygous mutation in the SCYL1 gene. He was admitted to the hospital twice by the age of two and five years due to acute hepatic failure triggered by a febrile illness. Drug-induced, infectious causes, and metabolic diseases were excluded. The liver function then gradually recovered. The patient had delayed gross motor development as he started to walk at 20 months of age. After the first episode of ALF, he had progressive difficulty in walking leading to frequent falls and ending with a complete inability to walk. A whole-exome sequencing (WES) test revealed that the patient has previously unreported autosomal recessive pathogenic non-sense variation c.895A>T (p.Lys299Ter) in exon 7 of the SCYL1 gene in a homozygous status. It is confirmed that the pathogenicity of this variant in the SCYL1 gene was associated with SCAR21 disease.

## Introduction

Acute liver failure (ALF) in childhood is a rare condition in which rapid deterioration of liver function results in altered mentation and coagulopathy in previously normal children [1]. It often affects young children and carries high morbidity and mortality [1]. The most prominent causes of pediatric ALF include drug-induced liver injury, infections, inherited metabolic diseases, autoimmune hepatitis, and genetic diseases [2]. However, about 54% of children younger than three years and 49% of all children have an indeterminate cause of ALF [2].

Spinocerebellar ataxia autosomal recessive 21 (SCAR21; OMIM #616719) is a very rare subtype of cerebellar ataxia caused by a mutation in the SCYL1 gene [3]. The disease phenotype is characterized by recurrent episodes of early childhood ALF, that are resolving but end with hepatic fibrosis [4]. In addition, children with this disease are affected by ataxia, peripheral neuropathy (PNP), mildly delayed motor development, and mild learning disability [3]. Upon extensive literature search, only 16 patients with SCYL1 have been reported worldwide [3-9]. Here, we report the first Bahraini child who presented with recurrent episodes of ALF in early infancy and was diagnosed with a novel homozygous mutation in the SCYL1 gene.

## Case presentation

A five-year-old Bahraini male was born at term via a normal vaginal delivery following an uncomplicated pregnancy for consanguineous parents. His birth weight was 2.46 kg and had an uneventful neonatal period. He was unknown to have any past history of chronic liver disease. He had bilateral club feet corrected with splints.

**Figure 1:**
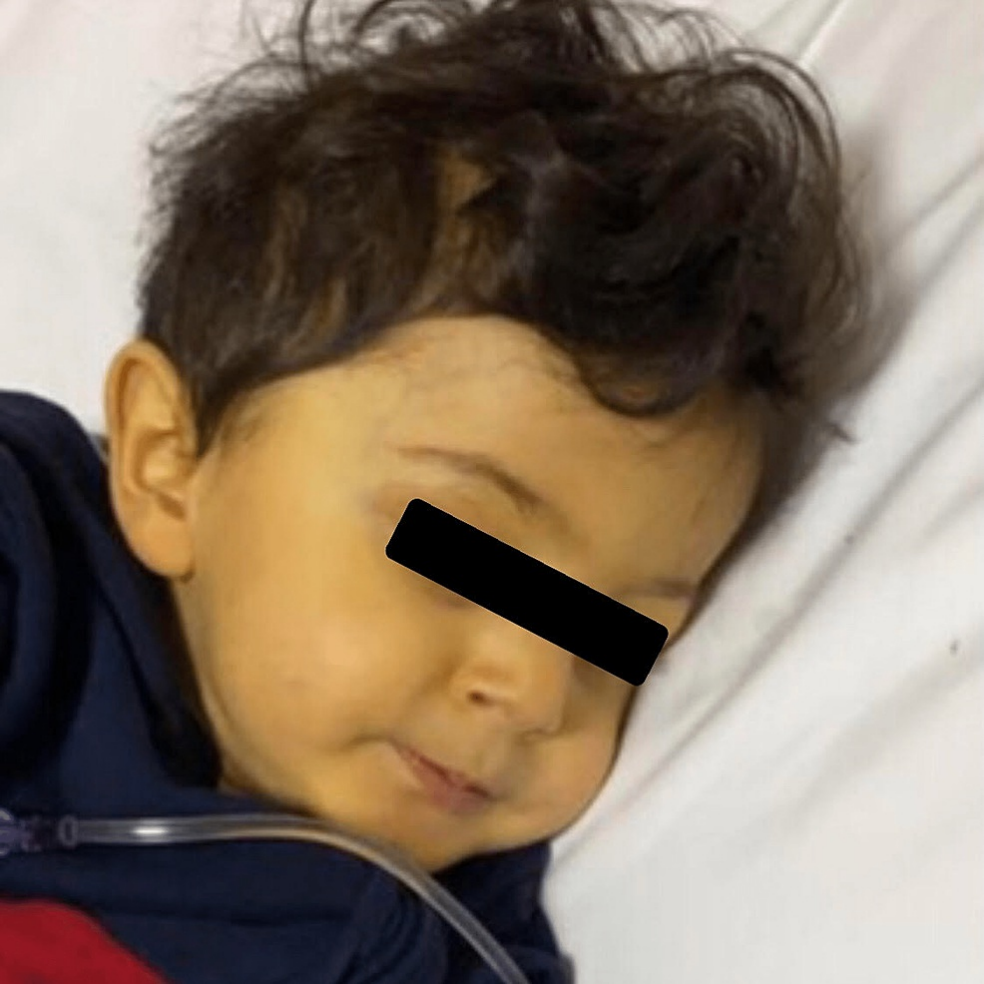
A two years and eight months old child with spinocerebellar ataxia-21 who presented with jaundice

The patient was vaccinated and up to date. He had a global developmental delay as he started to walk at 20 months of age, and started to talk at two years of age but he was still unable to draw vertical lines. At the age of 22 months, a sweat chloride test was done as an inquiry for failure to thrive, but the result was 39 mmol/L (normal range less than 35 mmol/L, and the level of more than 60 mmol/L is suggestive of cystic fibrosis). He had a healthy brother and sister with no family history of hepatic diseases. At two years and eight months of age, the patient presented with fever, vomiting, and yellowish discoloration of the skin and sclera (Figure 1). 

**Figure 2:**
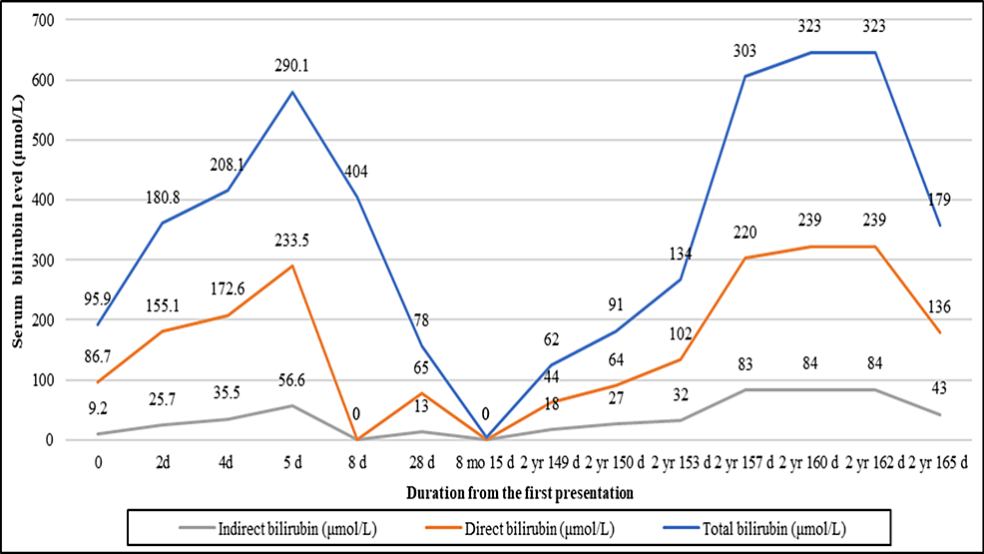
Levels of bilirubin in a male child with spinocerebellar ataxia-21

**Figure 3:**
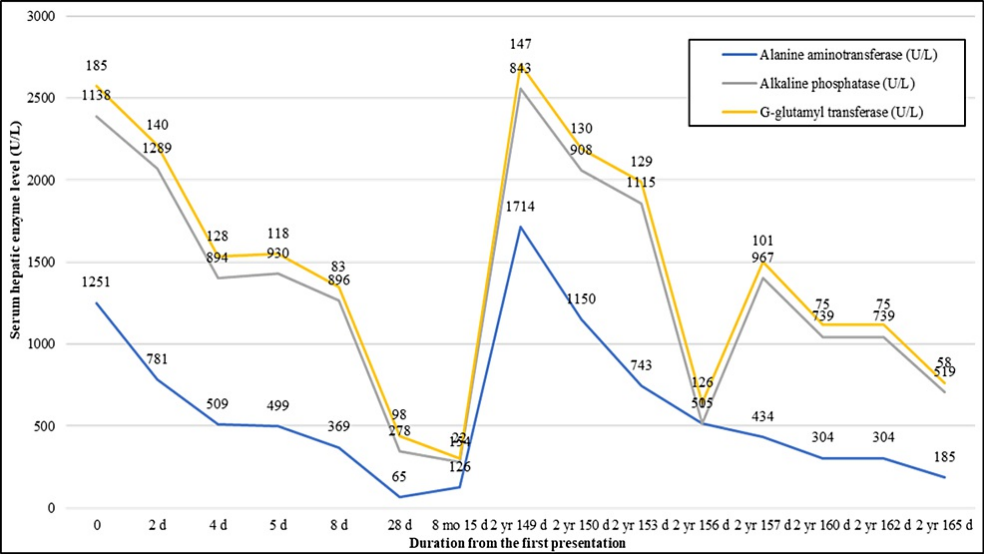
Levels of hepatic enzymes in a male child with spinocerebellar ataxia-21

**Figure 4:**
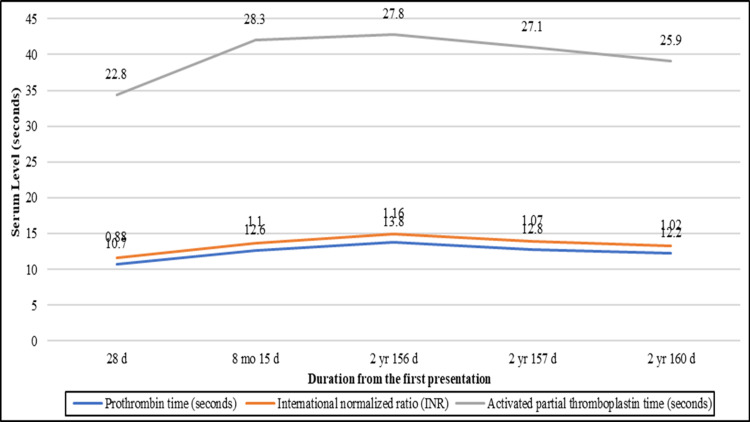
Coagulation profile in a male child with spinocerebellar ataxia-21

No history of hepatotoxic drugs or mushroom intake. His examination revealed a jaundiced child with hepatomegaly. He was admitted with a diagnosis of ALF (L-aspartate aminotransferase [ASAT]: 2845.7 UL; L-alanine aminotransferase [ALAT]: 1250.9 UL and international normalized ratio [INR] was not reported) (Figures 2-4). 

**Figure 5:**
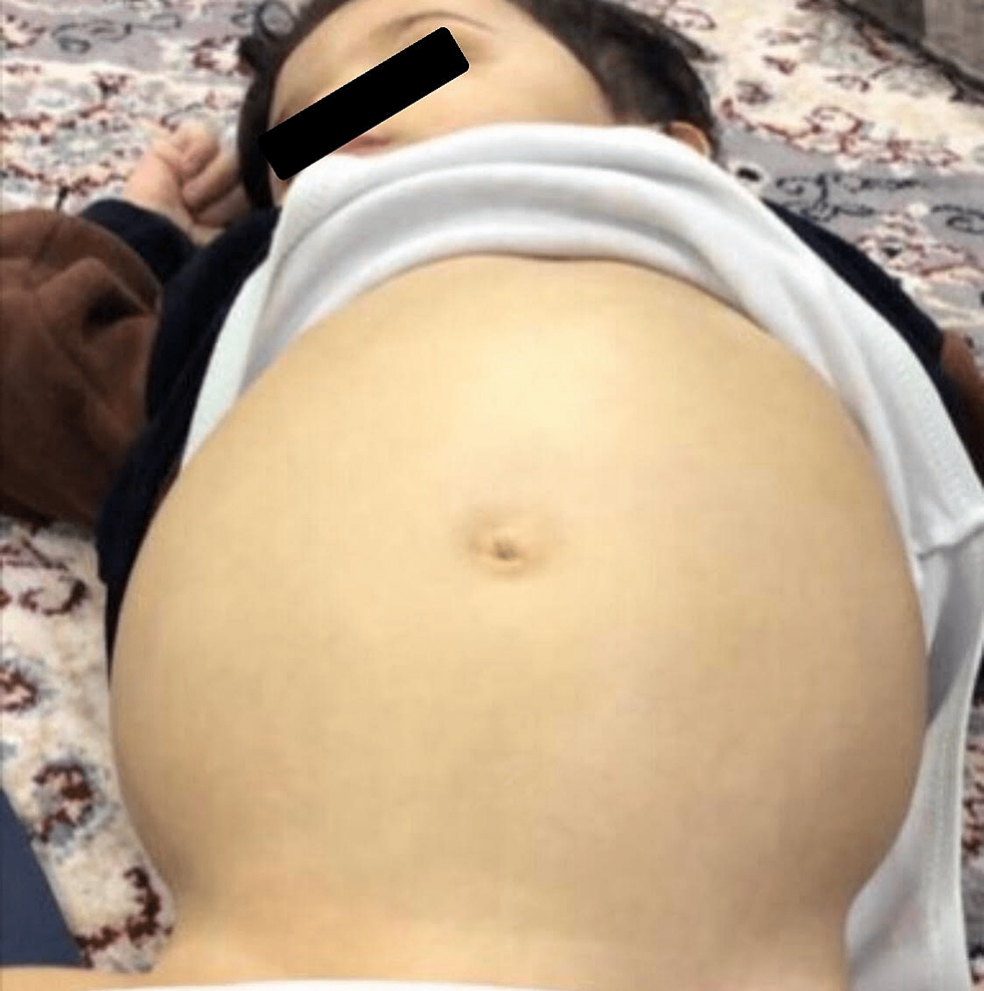
A two years and nine months old child with spinocerebellar ataxia-21 presented with abdominal distention secondary to acute liver failure and massive ascites

The patient was fully investigated for the possible causes of ALF and was found to have an acute cytomegalovirus (CMV) infection. Total Immunoglobulin (Ig) levels, auto-liver antibodies, serum copper and ceruloplasmin level were checked to rule out autoimmune hepatitis and Wilson disease, respectively as possible causes of his presentation. Serum and urinary copper and ceruloplasmin levels were within normal ranges. Eye examination was done and no evidence of Kayser-Fleischer rings. Abdominal ultrasound showed hepatomegaly with normal biliary system. Magnetic resonant imaging (MRI) did not reveal any changes of basal ganglia associated with Wilson disease. He was started on valganciclovir, ursodeoxycholic acid and omeprazole. Despite the treatment, the patient further deteriorated, and his liver functions were worsening. Accordingly, in view of high serum immunoglobulin M (IgM) level (2.26 g/L, normal range 0.50-2.20 g/L) and equivocally positive anti-smooth muscle antibodies, the patient was suspected to have an autoimmune hepatitis for which a trial of intravenous (IV) methylprednisolone was given as the liver biopsy was not done due to his coagulopathy. Despite the cause of liver failure was unknown, the option of liver transplantation (LT) was discussed with the parents as the patient's condition was worsening. However, as the patient showed improvement in liver functions, the patient was not sent overseas for liver transplant. Patient discharged home on the above-mentioned medications as oral syrups. Later, his father noticed that his child has a progressive difficulty in walking which led to frequent falls. This eventually progressed to complete inability to walk and was associated with intention tremors. Physical examination showed an icteric child with puffy face, lower limb edema and distended abdomen with ascites and hepatomegaly (Figure 5). However, there were no signs of motor or sensory neuropathy. Other systemic examinations were normal.

The patient was given albumin infusion with furosimide diuretic. Neurological assessment was performed by a pediatric neurologist (tone, power, reflexes and gait were all intact). However, he was unable to bear weight. Vitamin B12 and folate levels were normal. Electroencephalogram (EEG) was normal. Brain computed tomography (CT) scan neither revealed any evidence of acute ischemic or hemorrhagic insults, nor other significant findings associated with cerebellar changes were detected. MRI showed minimal periventricular white matter signal intensity, posteriorly adjacent to occipital horns of the lateral ventricle but not extending to the ventricle. Upon discharge, he was unable to neither walk nor stand up without support. One month following the discharge, his condition improved, and he was able to stand up and walk without support. However, he continued to have intention tremor.

The patient was admitted twice at the age of three years and four months and at the age of five years and one month with acute gastroenteritis episodes. In the first admission, the patient presented with recurrent episodes of high-grade fever and loose motions. This febrile illness was not associated with ALF episode (ALAT: 126 UL and INR: 1.1). He was admitted to the hospital for six days for IV hydration. He was discharged home after improvement. However, the second attack of acute gastroenteritis was associated with ALF (ALAT: 1714 UL and INR: 1.16). He presented again with a high-grade fever and diarrhea of six-day duration along with vomiting of three-day duration. This was associated with runny nose and productive cough. There was no history of contact with a sick patient. On physical examination, he was conscious, alert with icteric skin and sclera. He was vitally stable. His abdomen was distended with hepatosplenomegaly. All other examinations were unremarkable. An abdominal ultrasound showed mild hepatosplenomegaly with coarse hepatic echogenicity associated with mild dilation of intrahepatic duct, and mild gallbladder wall thickening, but no gallstone. Patient was evaluated for infectious, metabolic and immunological etiologies which led to a diagnosis of influenza A infection. He completed 10 days of cefotaxime antibiotic. He was discharged on prednisolone, ursodeoxycholic acid, and omeprazole.

On a follow-up visit, complete blood count, liver function tests, coagulation profile and CMV titer were within normal range. Moreover, repeated ultrasound was normal. As the patient presented with two episodes of ALF and neurological abnormalities, a whole-exome sequencing (WES) test was performed to evaluate for pathogenic variations. This revealed a previously unreported autosomal recessive pathogenic non-sense variation c.895A>T (p.Lys299Ter) in exon 7 of the SCYL1 gene in a homozygous status. Based on the ACMG classification, Varsome (https://varsome.com/) and Franklin (https://franklin.genoox.com/clinical-db/home) revealed that SCYL1 (NM_020680.4): c.895A>T (p.Lys299Ter) is a likely pathogenic variant. The literature review showed no report on this variant in the Clinvar (https://www.ncbi.nlm.nih.gov/clinvar/) and the frequency of this mutation is not available in gnomAD exome which supports the pathogenicity of this new mutation. Furthermore, the computational predictions such as MutationTaster, BayesDel, fitCons, and meta scores showed deleterious effects of this mutation. Notably, the length of the protein encoded by the SCYL1 gene is 808 amino acids, and the termination caused by the mutation as mentioned earlier makes a massive deletion after the K299 residue. Residues from 793 to 808 in the canonical protein sequence interact with COPB1 protein, which is deleted from the mutated allele of the current report.

Currently, the patient reached the age of five years and three months, but he had mild intellectual disability and speech delay. He is only able to say mama and baba and had a weak eye contact. However, he did not attend school yet, his parents stated that he is unable to write or read nor recognize colors, unable to dress himself independently and he cannot climb stairs without support.

## Discussion

SCYL1 gene is an evolutionarily conserved N-terminal protein kinase-like domain protein that has an important function in COP1-mediated retrograde protein trafficking in mammalian cells [1]. Loss of SCYL1 function can lead to neurodegenerative disorders in mice [1]. In human, an autosomal recessive variant in SCYL1 gene leads to a syndrome called SCAR21 [3]. This syndrome is characterized by early-onset episodes of ALF, cerebellar ataxia, and peripheral motor and sensory neuropathy [3].

Being an autosomal recessive disorder, sex is not an important factor in this type of inheritance. Nonetheless, our patient is a male child and according to the literature review, half of the 16 patients were males (50%). In our patient, both episodes of ALF were preceded by a febrile illness. Similarly, this finding has been noticed in all the reported cases [3-9]. Based on that, febrile illness is considered an important trigger for ALF episodes in patients with SCAR21 mutation.

In our patient, the first ALF episode was at the age of two years and eight months. Similarly, five of the reported cases experienced their first episode of ALF beyond the period of infancy, ranging from 14 months to four years [3,5,7,8]. On contrary, the majority of the reported cases had their initial presentation during infancy below the age of one year [3,4,6-9]. This variation in the timing of the first ALF episode might be attributed to either environmental factors such as the first episode of the febrile illness or the variability of the involved exon in the SCYL1 gene between patients as shown in Table 1 [3-5,7,9]. 

**Table 1:** Summary of previous studies on patients with spinocerebellar ataxia-21 along with our patient *The present study; ALF: acute liver failure; MRI: magnetic resonance imaging; CVA: Cerebellar vermis atrophy; ONT: Optic nerve thinning; M: Male; Yr: Years; Mo: Months; ID: Intellectual disability; ND: Not determined; F: Female

Study/year	Sex	Descent	Age at first episode	Gastroenterology		Development	Neurological symptoms				MRI		Exon of SCYL1 mutated	Zygosity
				No. of ALF episodes	Hepato-splenomegaly	Walking independently	Cognition	Gait ataxia	Spacticity	Tremor	CVA	ONT		
Isa et al. 2023^*^	M	Bahraini	2 yr	2	+	20 mo	Speech developmental delay & moderate ID	+	-	+	ND	ND	Exon 7	Homozygous
Campos et al. [5] 2020	F	ND	13 mo	1	ND	ND	Normal	+	ND	+	+	ND	Exon 12	Homozygous
Chavany et al. [6] 2020	M	ND	4 mo	2	Hepatomegaly	ND	Normal	-	-	-	-	-	ND	Heterozygous
Li et al. [7] 2019	M	Chinese	14 mo	4	+	ND	Low mental index	ND	ND	ND	-	-	Exon 1	Homozygous
Lenz et al. [8] 2017	3 M, 4 F	2 Germen, 2 Pakistani, 2 Turkish, 1 Italian	5, 6, 7, 10, 11, 18 mo & 4 yr	4 (n=4) 3 (n=2) 5 (n=1)	hepato-splenomegaly (n=4) hepatomegaly (n=3)	ND	Speech developmental delay (n=6) Mild MR (n=2) Stuttering (n=2) Lower end of the learning spectrum of the class (n=1) Intellectual borderline deficiency (n=1) Severe ID (n=1)	+ (n=2)	ND	+ (n=3)	+ (n=2) - (n=3) ND (n=1)	- (n=5) ND (n=2)	ND	ND
Shohet et al. [4] 2019	1 M, 1 F	Ashkenazi Jew	ND	05,Jan	Hepato-splenomegaly (n=1) Splenomegaly (n=1)	22, 12 mo	Mildly delayed (n=1) Learning disability (n=1)	+ (n=2)	-	+ (n=2)	- (n=2)	- (n=2)	Exon 4 (n=1) ND (n=1)	Homozygous Homozygous
Incecik et al. [9] 2018	M	Turkish	9 mo	2	-	24 mo	Mild learning disability	+	-	+	+	+	Exon 1	Homozygous
Schmidt et al. [3] 2015	1 M, 2 F	2 European, 1 Cuban	9 mo (n=2) 18 mo (n=1)	3 (n=2) 5 (n=1)	+ (n=3)	12, 17, 24 mo	Normal (n=1) Mild ID (n=1) Mild learning disability (n=1)	+ (n=3)	- (n=2) + (n=1)	+ (n=3)	+ (n=3)	+ (n=2) ND (n=1)	Exon 7 and 11 (n=2) Exon 9 and 12 (n=1)	Heterozygous for 2 mutations (n=2) Compound heterozygosity (n=1)

This patient had two episodes of ALF within two and a half years. The number of ALF episodes varies from one patient to another, ranging from one to a maximum of five episodes. Shohet et al. and Campos et al. reported one patient each who had only a single episode [4,5]. Yet, two episodes were reported by Chavany et al. and Incecik et al. in their case reports [6,9]. Three episodes were reported in two patients of Schmidt et al. and Lenz et al. each [3,8]. Four episodes were described by Li et al. and Lenz et al. [7,8]. Moreover, five episodes of ALF were described in case reports of Schmidt et al., Shohet et al. and Lenz et al. [3,4,8]. It is worth mentioning that the patient reported by Campos et al. has two genetic mutations (TRMU and SCYL1), both associated with acute hepatic insufficiency during infancy [5]. Contrary to what was explained in SCAR21 disease, the patient did not suffer from recurrent episodes of ALF, however, only experienced one episode with total recovery by the age of two-three years. This lower number of episodes is explained by the TRMU mutation, in which patients will suffer from early-onset liver failure with complete recovery by two or three years of age [5].

One of the findings in our patient was hepatosplenomegaly, which is similarly found in seven patients before [3,4,7,8]. Chavany et al. and Lenz et al. reported four patients with hepatomegaly, while Shohet et al. noted splenomegaly in one of his two patients [4,6,8]. However, neither hepatomegaly nor splenomegaly was found in the patient reported by Incecik et al. [9].

Regarding fine and gross motor development, our patient started to walk independently at the age of 20 months. Likewise, four of the previously described cases had delayed onset of walking, ranging from 17 months to 24 months [3,4,9]. Yet two other cases started to walk at the age of 12 months [3,4]. Data about the developmental milestones of the remaining ten cases were not reported.

In terms of the neurological symptoms, regarding cognition, our patient had a speech delay and mild intellectual disability which was similar to the majority of the reported cases [4,7-9]. However, Schmidt et al. and Chavany et al.'s studies revealed normal cognition in two out of the four reported patients (the only case and one out of the three cases, respectively) [3,6]. Moreover, our patient had gait ataxia that was in line with the eight patients reported by Shohet et al. and Lenz et al. [4,8]. Subsequently, the findings of our patient support the previous literature that cognitive impairment and gait ataxia are significant features of SCAR21 mutation. Furthermore, other neurological symptoms can be presented in patients with SCAR21 mutation, such as tremor and spasticity. Tremor was previously reported in nine patients while spasticity was noted in only one patient [3,4,8,9]. However, our patient had only tremor without spasticity.

The two most important findings on brain MRI in cases of SCAR21 are cerebellar vermis atrophy and optic nerve thinning. Of the 16 cases reported in the literature review, seven of the patients’ MRI showed positive cerebellar vermis atrophy, as reported by Schmidt et al., Lenz et al. and Incecik et al. [3,8,9]. While in three patients, the MRI showed positive optic nerve thinning as reported by Schmidt et al. and Incecik et al. [3,9]. However, these radiological findings were not documented in our patient which is similar to ten previously reported cases [4-8]. Accordingly, the SCAR21 disease should be thought of even in the absence of the classical MRI findings. The negative or not determined MRI findings can be explained by a lag of time between the clinical phenotype and imaging findings, and this stress on the importance of following up these patients and repeating MRI to assess for the development of pathological changes [4].

To sum up, there is a large phenotypic variability between patients from different families and even from a single family, as the two siblings of a young woman and her brother reported by Schmidt et al. [3]. In which each of them had different clinical features, depending on the type of mutation and its location in the gene [10]. Our patient had mutation in exon 7, that is similar to two patients reported by Schmidt et al. [3]. There is a wide range of variability in exons involved (exon 1,4,9,11,12) [3-5,7,9]. This wide range of variability in the location of the mutation within SCYL1 gene may explain the differences in phenotypes. It is mentioned that early variants have been suggested to cause a wider and more severe phenotype of the syndrome [8].

The management of our patient was mainly supportive and composed of IV fluid hydration, ursodeoxycholic acid, omeprazole, valganciclovir, antibiotics and prednisolone. Moreover, LT option was discussed with the parents, they were willing for it and the father was the candidate donor but it was not required as the patient was showing improvement both clinically and biochemically. This management was similar to most of the previously reported cases, in which their main treatment was supportive [4,6,7].

Although ALF is the main clinical feature of SCAR21 disease and LT can be a life-saving option, it was only reported in one out of the 16 patients. This was one of the seven cases reported by Lenz et al. who received LT at the age of 23 months, and the outcome has been satisfactory as the patient did not develop any further ALF episodes over a follow-up period of eight years [8]. Despite that LT can be an option for patients who are not responding to the supportive treatment and can carry a good outcome, the decision of LT should be taken with caution as most of the patients will respond to supportive care and ALF crises diminish with time [6]. Moreover, LT is not without risk, it can lead to serious complications, and will put the patient in an immunocompromise state for life [6].

## Conclusions

Although SCAR21 is considered a rare disease, children with fever-triggered recurrent ALF attacks should still have a genetic testing to exclude SCYL1 gene mutation as a cause, especially if it was accompanied by other neurological symptoms. Based on the literature review, it is concluded that the treatment of SCAR21 is still controversial, and the outcome of this disease is considered vague. Further studies are needed to cover all aspects of this genetic disease starting from presentation until prognosis, focusing on the optimal options for treatment and management.
